# Short-term Reactions Among Pregnant and Lactating Individuals in the First Wave of the COVID-19 Vaccine Rollout

**DOI:** 10.1001/jamanetworkopen.2021.21310

**Published:** 2021-08-17

**Authors:** Alisa Kachikis, Janet A. Englund, Michael Singleton, Isabela Covelli, Alison L. Drake, Linda O. Eckert

**Affiliations:** 1 Department of Obstetrics and Gynecology, University of Washington, Seattle; 2 Department of Pediatrics, Seattle Children's Research Institute, University of Washington, Seattle; 3 Institute of Translational Health Sciences, University of Washington, Seattle; 4 School of Medicine, University of Washington, Seattle; 5 Department of Global Health, University of Washington, Seattle

## Abstract

This cohort study investigates short-term reactions associated with COVID-19 vaccines among pregnant and lactating individuals vs individuals neither pregnant nor lactating but planning pregnancy.

## Introduction

Vaccines against SARS-CoV-2 are highly effective in preventing COVID-19 illness.^1,2^ Research has found that COVID-19 is associated with adverse events in pregnancy,^3^ and recommendations therefore include offering SARS-CoV-2 vaccines to pregnant and lactating individuals, despite their lack of inclusion in initial clinical trials.^4,5^ To date, limited data on vaccine and pregnancy outcomes exist for SARS-CoV-2 vaccines in pregnancy and lactation.^6^ The objective of this study was to investigate experiences of pregnant and lactating individuals after receiving COVID-19 vaccines.

## Methods

In January 2021, we launched an online prospective cohort study of adults primarily located in the United States who were pregnant, lactating, or planning pregnancy at the time of COVID-19 vaccination. This study was determined to be exempt from institutional review board review by the University of Washington (UW) Human Subjects Division (Common Rule category 2). We followed the Strengthening the Reporting of Observational Studies in Epidemiology (STROBE) reporting guideline. Individuals who were recruited and enrolled online in the UW COVID-19 Vaccine in Pregnancy and Lactation Registry using chain-referral and snowball sampling were invited to participate. Electronic written informed consent was obtained, and self-reported demographic (including race and ethnicity), pregnancy, vaccination perception, and outcome data, including report of day 1 vaccine reactions, were entered via surveys into Research Electronic Data Capture (REDCap) software version 11.1.2 2021 (Vanderbilt University). Race and ethnicity data were collected to report diversity representation within our study population. Options for race and ethnicity were outlined following the Centers for Disease Control and Prevention’s National Health Interview Survey race and ethnicity categories. We performed statistical analysis (ie, χ^2^ tests and 1-way analysis of variance, odds ratios [ORs], and 95% CIs) using Stata statistical software version 16.1 (StataCorp). An α level of *P* ≤ .05, 2-sided, denoted significance. Data were analyzed from January through March 2021.

## Results

As of March 16, 2021, 17 525 individuals (17 364 [99.7%] women among 17 418 individuals with sex data; mean [SD] age, 33.6 [3.6] years among 17 518 individuals with age data; 15 361 White individuals [87.6%] among all individuals) with known pregnancy status receiving at least 1 dose of a COVID-19 vaccine had enrolled in the study. Owing to missing data, percentages for participant characteristics are among those with data for that variable. There were 3 distinct groups: 7809 individuals who were pregnant (44.6%), 6815 individuals who were lactating (38.7%), and 2901 individuals who were neither pregnant nor lactating but planning pregnancy in the near future (16.5%) at the time of their first vaccine dose. Most individuals received the Pfizer-BioNTech BNT162b2 vaccine (10 790 of 17 431 individuals [61.9%] with data on vaccine type) or Moderna mRNA-1273 vaccine (6592 individuals [37.8%]). Most participants resided in the United States, were employed in health care, and had completed some college education (Table). Among all participants, 15 055 individuals (85.9%) reported receiving 2 doses.

**Table.** Baseline Characteristics Among 17 525 Participants^a^

Characteristic	Pregnant individuals (n = 7809)		Lactating individuals (n = 6815)		Individuals planning pregnancy^b^ (n = 2901)		*P* value
	Total^c^	No. (%)	Total^c^	No. (%)	Total^c^	No. (%)	
Vaccine							
Pfizer-BioNTech BNT162b2	7770	4777 (61.5)	6775	4156 (61.3)	2886	1857 (64.4)	.02
Moderna mRNA-1273		2970 (38.2)		2596 (38.3)		1026 (35.6)	
Janssen JNJ-78436735		23 (0.3)		23 (0.3)		3 (0.1)	
Dose 1 to survey completion, mean (SD), d	7565	25 (15.4)	6452	27.8 (14.8)	2833	31.2 (16.8)	<.001
Dose 2 to survey completion, mean (SD), d	6232	10.1 (10.6)	5863	11.1 (11.7)	2440	12.9 (11.9)	<.001
Age, mean (SD), y	7804	33.4 (3.6)	6814	34.1 (3.7)	2900	32.7 (3.5)	<.001
Gravidity, mean (SD)	5236	2.1 (1.3)	4614	2.1 (1.3)	1943	2.1 (1.3)	.56
Parity, mean (SD)	5245	1.2 (1.0)	4619	1.2 (1.0)	1948	1.2 (1.0)	.67
Trimester of pregnancy at dose 1							
First	7611	1822 (23.9)	NA	NA	NA	NA	NA
Second		3694 (48.5)		NA		NA	
Third		2095 (27.5)		NA		NA	
Race and ethnicity^d^							
American Indian or Alaska Native	7809	58 (0.7)	6815	67 (1.0)	2901	17 (0.6)	.09
Asian		640 (8.2)		489 (7.2)		183 (6.3)	.002
Black or African American		103 (1.3)		104 (1.5)		44 (1.5)	.53
Native Hawaiian or other Pacific Islander		34 (0.4)		32 (0.5)		7 (0.2)	.26
White		6802 (87.1)		5883 (86.3)		2676 (92.2)	<.001
Other^e^		118 (1.5)		68 (1.0)		45 (1.6)	.01
Hispanic ethnicity	7751	463 (6.0)	6761	444 (6.6)	2881	194 (6.7)	.21
Education							
Some college or less	7756	281 (3.6)	6761	377 (5.6)	2885	121 (4.2)	<.001
Bachelor's degree (eg, BA, AB, BS)		1929 (24.9)		1823 (27.0)		899 (31.2)	
Master's degree		2565 (33.1)		2342 (34.6)		1034 (35.8)	
Doctorate or professional degree		2981 (38.4)		2219 (32.8)		831 (28.8)	
Area of employment							
Health care	7633	5310 (69.6)	6539	4750 (72.6)	2812	2205 (78.4)	<.001
Academics or science		823 (10.8)		675 (10.3)		228 (8.1)	
Teaching or childcare		434 (5.7)		343 (5.2)		118 (4.2)	
Office work or tech industry		406 (5.3)		204 (3.1)		76 (2.7)	
Other^f^		660 (8.6)		567 (8.7)		185 (6.6)	
Influenza vaccine this last season	7755	7529 (97.1)	6776	6461 (95.4)	2878	2732 (94.9)	<.001
Tdap vaccine in current or recent pregnancy							
Yes	7662	1995 (26.0)	6755	6218 (92.0)	NA	NA	NA
Plan to get		59 (0.8)		NA		NA	
Don’t know		4698 (61.3)		138 (2.0)		NA	
No		910 (11.9)		399 (5.9)		NA	
Should individuals receive the COVID-19 vaccine?		If pregnant?		If lactating?		If planning for pregnancy?	
Yes	7456	6153 (82.5)	6466	6080 (94.0)	2669	2520 (94.4)	NA
Depends on circumstances		1300 (17.4)		382 (5.9)		148 (5.6)	
No		3 (0)		4 (0.1)		1 (0)	

Abbreviations: NA, not applicable; Tdap, tetanus, diphtheria, and acellular pertussis.

^a^ All variables are based on pregnancy status at enrollment.

^b^ Participants in the planning group were neither pregnant nor lactating.

^c^ Totals differ from overall group numbers owing to missing data for some individuals.

^d^ Categories are not mutually exclusive. Missing numbers indicate participants who selected “Prefer not to answer.”

^e^ Participants could select other if they did not choose outlined race and ethnicity categories or in addition to other race and ethnicity categories. Those who selected other were given the opportunity to specify their own category.

^f^ Other includes military personnel, first responders, and individuals working in agriculture, manufacturing, construction, service, hospitality, retail industries, and other areas of employment.

Among all participants, 17 005 individuals (97.0%) reported any postvaccination reactions after the first dose, with the most common reactions being pain at injection site (16 019 individuals [91.4%]) and fatigue (5489 individuals [31.3%]). The frequency of reactions after the second dose was higher than after the first dose (eg, 10 399 individuals [69.2%] with fatigue after the second dose), but with similar distribution of symptoms (Figure). Odds of several reactions were statistically significantly decreased among individuals who were pregnant (eg, fever after BNT162b2 dose 2: OR, 0.44; 95% CI, 0.38-0.52; *P* < .001 and after mRNA-1273 dose 2: OR, 0.48; 95% CI, 0.40-0.57; *P* < .001) compared with individuals who were neither pregnant nor lactating (Figure). Mean (SD) maximum self-reported temperature was 38.1 (0.6) °C (100.6 [1.0] °F) among 499 participants with fever after dose 1 (including 131 pregnant individuals) and 38.2 (0.6) °C (100.7 [1.0] °F) among 3293 participants with fever after dose 2 (including 1051 pregnant individuals). Participants seeking medical care after vaccination included 100 individuals (0.6%) after dose 1 (including 50 pregnant individuals) and 221 individuals (1.5%) after dose 2 (including 156 pregnant individuals).

**Figure.**
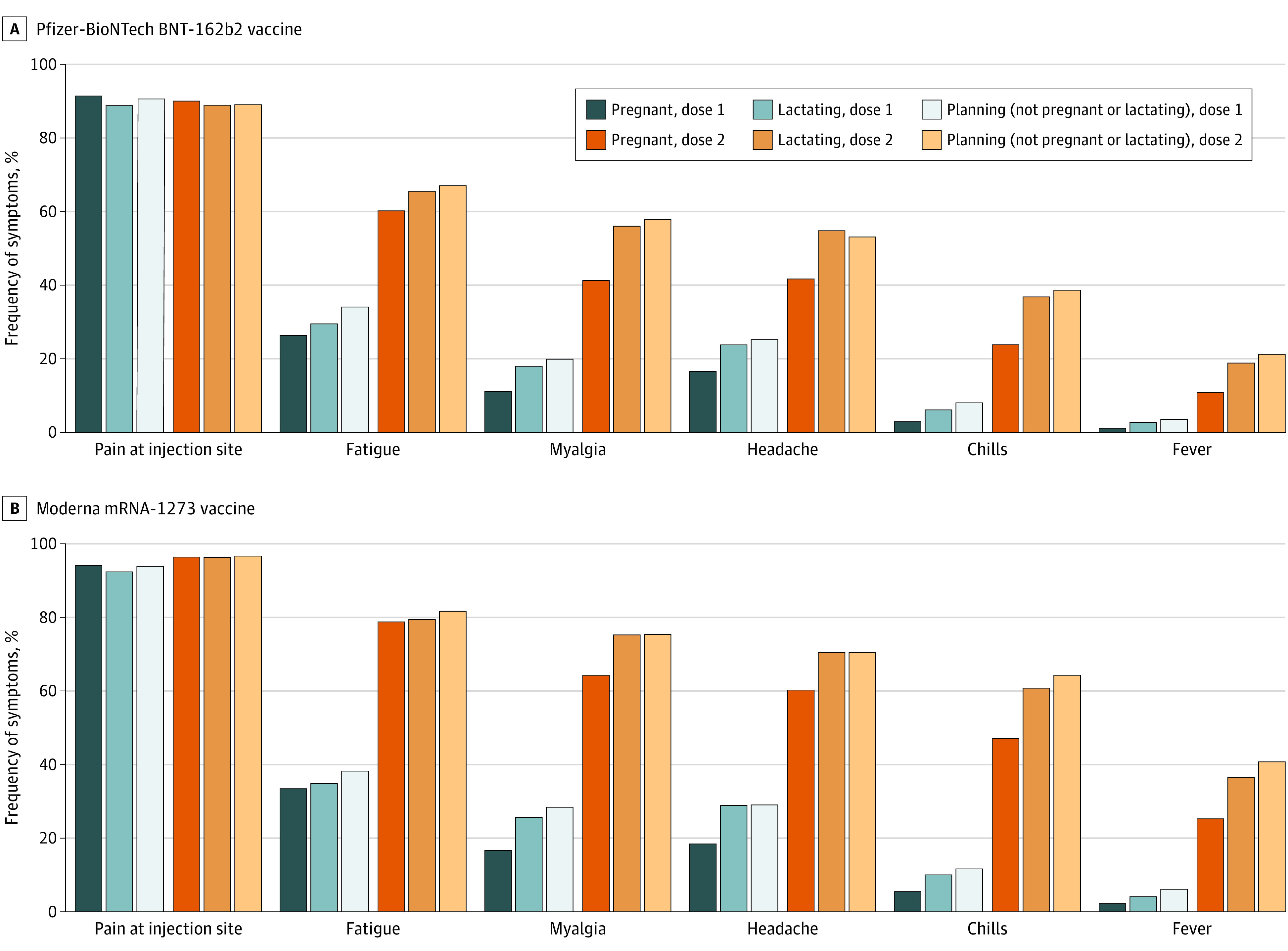
Reactions 1 Day Postvaccination

Among pregnant participants, any obstetrical symptoms were reported by 346 of 7809 individuals (4.4%) after the first dose and 484 of 6444 individuals (7.5%) after the second dose. Altogether, 6586 pregnant individuals (84.3%) had reported a second vaccine dose at the time of data analysis. Of these, 6244 individuals (94.8%) were still pregnant, while 288 individuals (4.3%) had delivered and 49 individuals (0.7%) reported miscarriages at the time of their second vaccine dose. Among lactating individuals, interrupted breastfeeding after vaccination was reported by 155 of 6815 individuals after the first dose (2.3%) and 130 of 6056 individuals after the second dose (2.2%), decreased milk supply for less than 24 hours by 339 individuals after the first dose (5.0%) and 434 individuals after the second dose (7.2%), and concerns about the infant after vaccination by 208 individuals after the first dose (3.0%) and 267 individuals after the second dose (4.4%).

## Discussion

This large prospective cohort study found that COVID-19 vaccines were well-tolerated among individuals who were pregnant, lactating, or planning pregnancy. A strength of this study was the ability to compare vaccine reactions and perceptions in pregnant and lactating individuals vs individuals of similar age and fertility intentions who were neither pregnant nor lactating. Vaccination reactions for day 1 were similar among groups and comparable with findings among pregnant individuals previously reported.^6^ All groups reported increased reactions following dose 2 of BNT162b2 and mRNA-1273 vaccines.

Study limitations include that participants were drawn from a convenience sample with self-reported reactions and with limited perinatal outcome assessment, reflecting the first wave of vaccination, which largely consisted of health care workers owing to vaccine eligibility at the time of this ongoing study. As a result, our findings may be biased and not generalizable to all populations. In addition, there is potential participant overlap between our study and similar studies.^6^ Further studies are ongoing to investigate outcomes after receipt of COVID-19 vaccines among pregnant and lactating individuals.
